# “Constantly overwhelmed and desperate for help”: Parents’ experiences of supporting their autistic child with mental health difficulties in the United Kingdom

**DOI:** 10.1371/journal.pmen.0000377

**Published:** 2025-09-22

**Authors:** Emma Ashworth, Lucy Bray, Claire Hanlon, Georgia Pavlopoulou, David Moore, Bethany Donaghy, Elizabeth Coen, Harvey Stanway, Ellen Firth

**Affiliations:** 1 School of Psychology, Liverpool John Moores University, Liverpool, United Kingdom; 2 Faculty of Health and Social Care, Edge Hill University, Ormskirk, United Kingdom; 3 Group for Research in Relationships And NeuroDiversity, Department of Clinical, Educational and Health Psychology, University College London, London, United Kingdom; 4 Anna Freud Centre, London, United Kingdom; PLOS: Public Library of Science, UNITED KINGDOM OF GREAT BRITAIN AND NORTHERN IRELAND

## Abstract

Autistic children and young people are at increased risk of experiencing mental health difficulties, but often face delays or barriers to accessing support. While evidence exists regarding parents’ experiences of supporting an autistic child, there is a lack of focus on parenting autistic children who are also experiencing mental health difficulties. This is despite the high likelihood of co-occurrence, the increased complexity this can bring, and the potential impact on the parents and their children. Thus, the present study aimed to explore parents’ experiences of supporting their autistic child with mental health difficulties in the United Kingdom (UK). Mixed-methods surveys were completed by 300 parents/carers of autistic children who had previously sought help for their child’s mental health difficulties. Qualitative data were extracted from open-text questions pertaining to parents’ perceived impact of their children’s mental health difficulties on all aspects of their life. Data were analysed using reflexive thematic analysis. Three themes, along with associated subthemes, were identified, namely 1) Deteriorating parental wellbeing, 2) The knock-on effect on the whole family, and 3) A lack of support. Findings underscore the significant challenges faced by parents, as they were often left to manage their child’s deteriorating mental health without sufficient professional support. The emotional and physical toll on parents was thought to be exacerbated by long waiting times, inadequate support services, and a lack of understanding of autism within healthcare and educational systems. There is an urgent need for more neuroaffirmative, personalised approaches to supporting autistic children and their families, along with timely access to effective interventions. By ensuring earlier intervention and reducing systemic barriers, both the mental health of children and the wellbeing of their families can be significantly improved, ultimately fostering better outcomes for all involved.

## Introduction

Autism is a neuro-developmental difference in the way the brain develops, meaning that autistic people may process sensory information differently, have different cognitive profiles, and use different communication styles to neurotypical individuals. Recent years have seen a significant increase in the number of children and young people (CYP) receiving an autism diagnosis [1], alongside growing recognition of the mental health challenges that many autistic individuals face. Indeed, autistic CYP experience a higher prevalence of mental health difficulties, with up to 80% experiencing a mental health condition compared to 12% in the general population in the UK [2,3]. Similarly, autistic CYP are almost twice as likely to think about or attempt suicide than neurotypical CYP, with one-fifth experiencing suicide ideation [4], and are also more likely to present at Emergency Departments or be admitted to hospital for suicidal crisis [5,6].

The prevalence of autistic CYP who meet the criteria for a mental health condition has prompted the National Institute for Health and Care Excellence (NICE) to provide guidance on best practices for health and social care services working with autistic CYP. The NICE Autism Quality Standard [7] specifies that CYP referred for a diagnostic assessment of autism should receive their evaluation within three months, and any co-existing physical or mental health conditions should also be assessed. Additionally, the guidelines emphasise that mental health professionals must possess knowledge of autism to comprehend how its traits influence the treatment of concurrent mental health conditions. When necessary, professionals are encouraged to consult with a specialist autism team for advice on the implementation and modification of interventions. Despite this existing guidance, many families face challenges in obtaining suitable care and support for their child’s co-occurring mental health issues via Child and Adolescent Mental Health Services (CAMHS) [8].

In the UK, CAMHS is operated by the National Health Service (NHS) for CYP up to age 18 (although some support individuals aged up to 25), which is free at the point of use. CAMHS offers support for a range of mental health difficulties, as well as assessments for neuro-developmental differences such as autism. CYP need to be referred to CAMHS for support, typically via their doctor or school, although parents/carers can also self-refer. However, waiting times for a first appointment and to be allocated a case worker can be long. This is thought to be due to decades of under-investment and under-resourcing of the NHS as a whole, as well as CAMHS more specifically, with only 8% of the mental health budget and less than 1% of the total NHS budget spent on CYP’s mental health [9,10].

Findings from our recent study [8] demonstrate the ongoing struggles that parents face when seeking professional help from CAMHS for their child in the UK. Parents reported that their children were not referred to CAMHS or were rejected without an assessment, often due to issues relating to diagnostic overshadowing (whereby mental health needs are overlooked in assessments, as symptoms are conflated with traits relating to autism), a high threshold for assessment, or a lack of professional knowledge about autism and care pathways. Those who were referred reported a lack of reasonable adjustments and offers of ineffective or inappropriate therapies, leaving CYP unable to engage, and thus not benefitting. Ultimately, parents felt their child’s mental health difficulties either did not improve or declined to the point of crisis.

In addition to the difficulties experienced by CYP themselves, being the parent/carer of an autistic child can bring about a unique set of challenges [11], with the co-occurrence of their child’s mental health difficulties further complicating the caregiver experience. This is particularly the case in the current UK climate, given the aforementioned issues in CAMHS preventing access to effective and timely support [8]. However, despite the high prevalence of mental health difficulties in the autistic population, there is limited research exploring the experiences of parents supporting an autistic child with mental health difficulties, and the perceived impact on their own wellbeing. Understanding parents’ lived experiences can help to inform the implementation of appropriate policies, practices, and support services, improving both CYP and parents’ mental health and ensuring that funding and resources are allocated effectively.

One concern for parents is the impact that supporting an autistic child can have on their own emotional and psychological wellbeing when living in a society where support is limited or is designed for neurotypical individuals. Research has highlighted that parents of autistic CYP can experience higher levels of stress and are at increased risk, relative to parents of neurotypical children, of financial strain, and poorer physical and mental health [12]. Indeed, a systematic review of 12 studies conducted by Vasilopoulou and Nisbet [13] consistently identified poorer quality of life among parents of autistic CYP, compared to those of neurotypical CYP; most parents demonstrated lower subjective physical and mental health as well as poorer social functioning and lower satisfaction with their environment. Factors affecting quality of life included household income, employment status, being a mother, social support, and support from services, as well as child-level factors such as externalising/conduct difficulties. However, all articles included in this review were from outside the UK, and so the experiences of UK-based parents are not known. Studies were also quantitative in nature, limiting the degree to which parents’ personal experiences could be explored.

One meta-synthesis that did include qualitative data was conducted by Ooi et al. [14]. Across 50 international studies, parents described increased stress and an impact on their daily lives while trying to meet their child’s needs, with reports of emotional and physical consequences, strain on their marriages, and isolation and stigma. The personal lives of parents were also affected in terms of their career, leisure time, and health, and parents noted a lack of support from services. However, parents also expressed the joy they found in raising their child and felt that it promoted positive characteristics in themselves (e.g., compassion, appreciation). These themes were mirrored in other qualitative reviews by DePape and Lindsay [12], Corcoran et al. [15], and a more recent meta-synthesis by Samsell et al. [16], although Corcoran et al. and Samsell et al. focused exclusively on US parents.

While there is clear evidence that being a parent of an autistic child can impact their physical and psychologically wellbeing, both positively and negatively, the vast majority of the research to date has focused on the experiences of parenting autistic CYP generally. Research has traditionally also adopted a ‘within-child deficit’ approach, rather than focusing on the systems within which the child and their families reside, and their experiences of navigating these. There is a paucity of evidence regarding parents’ experiences of supporting autistic CYP specifically with mental health difficulties, and particularly in attempting to access community mental health support. Indeed, only one study [17] has explored this issue, and while it provides valuable insights into UK parents’ experiences, it was conducted 15 years ago – the time elapsed since then has seen changes in diagnostic procedures, social norms, the process of applying for Education, Health and Care Plans (EHCPs), and in the funding and delivery of healthcare services [14]. Thus, findings may not be reflective of current parents’ experiences.

## The current study

While evidence exists regarding parents’ experiences of supporting an autistic child, there is a lack of focus on parenting autistic CYP who are also experiencing mental health difficulties, despite the high likelihood of co-occurrence, the increased complexity this can bring, and in light of the current issues regarding the accessibility of child and adolescent mental health services. It is vital to understand the current experiences of parents if appropriate and effective support is to be provided to families. Thus, the present study aimed to explore UK parents’ experiences of supporting their autistic child with mental health difficulties. While the focus of this study is the experiences of UK parents, it is possible that many of the findings, and implications of these, may be transferable to other countries worldwide.

## Methods

### Design

A qualitative design was used for this study. This paper reports on the open-text data collected as part of a larger, mixed-methods survey [8] examining parents’ perceptions of the accessibility of CAMHS for autistic CYP. Data were extracted from four open-text questions pertaining to parents’ perceived impact of their child’s mental health difficulties on all aspects of their life.

### Ethics

Ethical approval was provided by Liverpool John Moores University’s Research Ethics Committee (ref:23/PSY/046). Formal opt-in consent was obtained electronically (via tick-box at the beginning of the survey).

### Public involvement

The team engaged in consultation with autistic adults, autistic CYP, and parents of autistic CYP, to inform all stages of the study. This is reported according to the GRIPP-2 short-form in Table 1.

**Table 1:** GRIPP-2 short form for reporting public and patient involvement [18].

Section	Overview
Aim	To ensure that the study addressed issues of importance to parents and autistic CYP, that materials were accessible, and that reporting of the findings were clear.
Method	Two parents and three autistic CYP were consulted via Teams, and through sharing iterations of the survey questions for feedback. Three autistic young adults with lived/living experience were involved in developing the study and drafting the paper as co-authors.
Outcome	Consultations resulted in survey questions being amended in format (e.g., questions organised into stages of accessing CAMHS) and response options (e.g., more open-text options). Consultation also informed the recruitment information (e.g., the design of the study flyers and the language used, including noting that we are ‘a team of autistic and non-autistic researchers’) and networks used for recruitment.
Discussion	Consultation was important in ensuring the survey addressed issues of importance to parents and was not too onerous. The lived experience of team members was important throughout analysis/writing.
Reflection/Critical Perspective	The team ensured an open space for team members with lived/living experience to reflect on and be supported when analysing what were often distressing and challenging accounts.

### Participants

Parents were recruited using volunteer/opportunity sampling, via social media (e.g., Facebook and X) and through contact with relevant organisations and networks. Parents were invited to take part if they lived in the UK and had an autistic child (diagnosed, self-diagnosed, or on the pathway for diagnosis – see Table 2) who had experienced mental health difficulties, for which they had sought professional help in the last five years.

**Table 2:** Participant demographic detail.

Demographic	n (N = 300)	Percentage
**Region**		
England	263	87.7
Scotland	17	5.7
Wales	16	5.3
Northern Ireland	4	1.3
**Child’s Gender**		
Girl	144	48.0
Boy	129	43.0
Non-binary	12	4.0
Transgender	11	3.6
Prefer not to say	2	0.7
**Child’s Autism Diagnosis**		
Formal diagnosis	240	80.0
Self-diagnosed	18	12.0
On a waiting list	38	12.7
**Child’s Education Setting**		
Mainstream	166	55.3
Special/alternative provision	66	22.0
Home-schooled	20	6.7
Flexi/online	8	4.3
Other	9	3.0
Not in school	29	9.7

N.B. where percentages do not add up to 100%, there are some responses missing.

300 parents/carers participated. The majority (87.7%; n = 263) lived in England. Their children were aged between 5 and 25 years (mean age = 13; SD = 3.15), 48% (n = 144) were girls, 43% (n = 129) were boys, and 7.6% (n = 23) were non-binary or transgender. 84.7% (n = 253) also had another long-term physical health condition (e.g., hypermobility, genetic conditions) or neurodevelopmental difference (e.g., ADHD, dyspraxia, dyslexia). Table 2 provides further detail.

### Data collection

A mixed-methods survey, dominated by exploratory qualitative questions, was developed for this study [19]. The survey was available online via QuestionPro (https://eu.questionpro.com/) between 30^th^ June and 16^th^ October 2023. The surveys could be completed at a time and place to suit participants. The survey included open- and closed-questions grouped into sections. Four questions pertaining to parents’ perceived impact of their child’s mental health difficulties were used for the purposes of this paper (“What mental health difficulties first led to you/your autistic child seeking help from mental health services?”, “How do you feel these mental health difficulties impact on your child’s daily life?”, “How do you feel these difficulties impact on your daily life and the life of your family?”, “Do you think your child’s ability to attend or engage with education/school has been impacted by their mental health difficulties? If yes, please explain how”). Surveys began with opt-in consent and demographic questions, as well as an introduction to the research team, before exploring parents/carers experiences.

### Analysis

Qualitative responses were extracted, collated in Microsoft Word, and then transferred to NVivo 11 for analysis. Responses were analysed using reflexive thematic analysis [20]. EA, LB, CH began by reading and rereading through the responses, familiarising themselves with the data. Data were then coded and subsequently collated into potential themes. Next, these potential themes were shared with the other researchers in the team for feedback and reflexive discussion, and were subsequently refined and defined. Further analysis then occurred via the process of writing (Smith, 2015), before being shared again with researchers for more feedback. Our design was informed by Yardley’s (2000, 2015) quality principles of (1) sensitivity to research context, (2) commitment and rigour, (3) transparency and coherence and (4) impact and importance. The Consolidated Criteria for Reporting Qualitative Research (COREQ; Tong et al., 2007) was utilised when describing methods and presenting the findings [21].

### Reflexivity

We acknowledge how our lived experiences and interests may have influenced our approach and interpretation. We have academic and professional experience from our work within the fields of health, psychology, counselling, and special educational needs and disability (SEND) provision, as well as personal and/or family experience. The team is made up of neurodivergent and neurotypical individuals, and parents of neurodivergent children. In line with a reflexive approach, we did not aim for ‘accurate’ or ‘reliable’ coding (Byrne, 2022) or the use of rigid coding frameworks when analysing the qualitative data. When discussing findings, the team reflected on the assumptions and expectations they brought to the work, as well as how their own experiences may have influenced their interpretations of the data.

## Findings

Findings from the thematic analysis identified three themes, along with associated subthemes. These are presented in Table 3.

**Table 3:** Themes and associated subthemes.

Themes	Subthemes
“You are only as happy as your unhappiest child”: Deteriorating parental wellbeing	Worry, exhaustion, and distress
	Providing support is all encompassing
“The devastating impact on us as a family”: The knock-on effects on the whole family	Living in a tense home
	Strained relationships
“Isolated, alone, exhausted, and unsupported”: A lack of support	Loneliness, isolation, and stigma
	The battle for more effective support from services

To provide further context regarding their children’s mental health difficulties, parents were asked to describe these at the beginning of the survey. Responses were coded and the number of code occurrences counted. The most frequently cited difficulty was anxiety (n = 154), followed by school-based anxiety or difficulties at school (n = 96), and suicide ideation (n = 83). Table 4 provides an overview of each response type.

**Table 4:** Parents’ description of mental health difficulties experienced by their child.

Type of Difficulty Coded	Frequency of Code	Illustrative Quote
Anxiety	154	“Constant crying, worrying, scared, panicking all the time, screaming, separation anxiety, most of all feeling and looking sad” (P48)
School-based anxiety/difficulties	85/11	“Severe anxiety (possible PTSD) from experiences at first high school which left her feeling suicidal and making some attempts to act on those feelings” (P39)
Suicide ideation	83	“She told me herself she wanted to die because of her worries” (P120)
Depression	68	“Went from happy, intelligent child to depressed and self-harming” (P22)
Self-harm	55	“Trying to strangle himself, threatening to stab himself, self-harm, banging his head at school” (P63)
Violence, aggression, or anger	28	“Angry, explosive behaviour, hurting others and smashing up things” (P296)
Difficulties with eating	24	“Non-functioning day-to-day, shut down, not speaking, eating, or getting up from bed” (P180)
Difficulties with regulation	21	“My son was having a difficult time regulating himself in school resulting in him having severe anxieties and talking about suicidal ideation” (P153)
Difficulties socialising or isolation	20	“School refusal...stayed in bedroom, whole routine changed... wouldn’t talk or speak or engage with anyone” (P57)
Distress/breakdowns	19/18	“She had a mental breakdown. Stopped eating, sleeping and withdrew into herself” (P13)
Sleep difficulties	16	“She couldn’t settle at night so it was a vicious cycle of tiredness, anger, upset and self-harm” (P284)
Low self-esteem	15	“My child spoke about hating himself, that nobody understood him and that he felt the world would be a better place if he wasn’t in it” (P263)
Obsessive thoughts/behaviours	15	“OCD became so controlling she couldn’t leave the house” (P26)
‘Meltdowns’	15	“Difficulties with meltdowns and seeming to understand situations differently to the reality” (P239)
Distress following trauma	14	“My child went on to develop severe anxiety as a result of school based trauma” (P37)
Symptoms of psychosis	8	“Apparent psychosis - hearing voices, being told to kill others, wandering streets at night looking for a child they could hear in distress” (P241)
Speech & language difficulties (mutism)	8	“Anxiety and selective mutism following school-based trauma” (P267)
Intrusive thoughts	7	“He had many intrusive thoughts telling him he was stupid and didn’t deserve to be alive and was a burden on everyone. He believed these” (P28)

1. **“You are only as happy as your unhappiest child”: Deteriorating parental wellbeing**
  1.1. ***“I have held all the risk”: Parental worry, exhaustion, and distress***

Many parents commented on the perceived impact that supporting their child had on their own mental health, as they were caring for their child’s significant mental health needs with little or no external support. Multiple respondents highlighted the anxiety they experienced due to managing the mental health concerns of their autistic child alone, describing the worry as “inexplicable” (P281): “it has become all consuming. I think and worry about his mental health daily and how we move forward” (P196). They also found it “upsetting to see my child distressed” (P 138): “my smiley little girl has become a pale, tired, stressed little lady” (P103). Parents found this particularly difficult when they did not feel they could help: “it was awful. Watching him in such distress but not being able to help him was deeply painful” (P37).

They often attributed their deteriorating wellbeing to the “immense and unsustainable amount of pressure” (P30) that they felt, particularly around keeping their child safe from self-harm, suicide, or other behaviours of concern that resulted from the distress they were experiencing: “constant anxiety that he would harm [him]self. Taking objects and materials out of his room... locating medication to alternative location” (P216). One parent described how concerns for their child’s safety during a mental health crisis and managing the associated risk had resulted in them becoming exhausted:

“I’m very very tired, I’ve had to advocate, lead, hold my child in the middle of the night to prevent death. I have to lock everything away, I have to monitor money, food, washing, I live day by day, hour and occasionally minute by minute in fear of the calls, the breakdowns, the desire they will eat, sleep, go to school, get home alive. I have held all the risk.” (P41)

Worries about safety were particularly prevalent overnight, resulting in parents missing out on sleep to watch their child: “exhausting myself through the night to ensure she’s ok” (P46). Parents’ sleep was also impacted because of their child’s sleep problems; “we generally stay up with our daughter until she goes to sleep” (P4), resulting in them adopting new sleeping arrangements: “he now co-sleeps with me so my sleep is impacted too” (P197). This was similarly the case for siblings in the home, who were also woken at night or prevented from sleeping: “his sibling’s sleep is also affected on days he has meltdowns at night” (P298).

Many parents described how they were “exhausted”, often due to the time required to support their child, “exhausting having to regulate from constant meltdowns” (P147), worrying about their child’s difficulties with school, “it’s exhausting as I cannot relax at any school event/concert. Don’t even mention sports day.” (P70), and trying to access help and respite from services, “we are at breaking point unable to get the help we desperately need” (P268). Ultimately, numerous parents indicated experiencing mental health issues themselves while awaiting formal support for their child’s mental distress, with some experiencing “panic attacks for the first time” (P161) and others needing medication: “I had multiple periods off sick due to the stress and anxiety. Was on antidepressants myself for nearly 3 years and still have difficulty motivating myself to do anything for myself as often feel exhausted and burnt out” (P95). In addition, several respondents also noted how their physical health had declined as a result of their poor mental health: “it’s affected my physical health and contributed to my chronic fatigue” (P30).

1.2. ***“This is not a life, this is not living”: Providing support is all encompassing***

Several parents described how their lives have been “taken over” (P266) by trying to support their child’s mental health without effective provision from services, describing it as “all encompassing” (P164). Parents felt that the impact of caring for their child’s mental health difficulties without any support was “totally life changing” (P69), meaning that they did not have any time for themselves, activities, or socialising, and that their “life has become very small” (P143). Parents noted how they did not get “enough rest/downtime” (P235), had “no opportunity for self-care” (P197), and “barely leave the house” (P261). One parent described: “My entire life is now centred around her. I am unable to work, unable to sleep and often cannot leave the house. I have no hobbies or free time. I can’t even watch TV” (P106).

Often, their time was dedicated to comforting their child or providing them with the extra attention they needed due to their mental health difficulties: “she will not leave my side a lot of the time and needs constant reassurance that she will be ok” (P120). This “separation anxiety” (P121) was commented upon regularly, with parents describing how it “places severe limitations on my life and family life in general” (P198). Reassurance for children’s anxieties was also commonly described by parents: “we spend almost all our waking time reassuring him about his various worries or physical symptoms” (P174).

To help manage their children’s mental health (particularly anxieties), many parents engaged in significant forward planning, anticipating what their child would or would not be able to manage, and making adjustments to plans pre-emptively: “everyday we have to work out what will and won’t be ok for my daughter” (P120). They also often invested time in activities to help their child regulate their emotions and to sleep: “much of my time is spent trying to calm my child. My evenings are often spent trying to help my child sleep” (P84). Parents also reported spending large amounts of time trying to prevent their child from harming themselves: “every day feels a challenge to get through - to try and support and keep her living” (P300).

When children’s mental health was poor, parents described actively trying to ensure it did not deteriorate further: “these difficulties have almost taken over our daily life now because as his parents we are constantly trying to keep his mood from slipping dangerously low and trying to evaluate when it is” (P266). Poor mental health often led to developmental regressions, requiring parents to provide personal care for their child, in addition to implementing strategies to keep them safe from harm. One parent summarised all of these issues:

“We need to go everywhere with her in case she attempts suicide or just has a panic attack... We cannot have medicine in the house (so if another child is ill, we just hope it’s not in the middle of the night and can get medication), and we cannot have scissors, razors, or pencil sharpeners in the house. All kitchen knives are in a safe. We have alarms set every 3 hours so we can check on her during the night, and will often need to sit with her for some of these hours, while she cries. She is depressed and doesn’t care for herself, so we shower her and help her during period times. She also refuses to eat some days, so we have to force her to eat small amounts during those days… we are bumbling along the best we can.” (P251)

The impact on parental employment was commented upon frequently, with many parents missing work, “made mum and dad miss lots of work or be late for work” (P187), reducing their hours, “had to massively reduce my working hours to be at home full-time” (P195), or giving up their jobs entirely, “I’ve had to leave employment and university to be a full-time carer” (P260). This was generally due to their child not wanting/being able to attend school, or not wanting to leave the house entirely, largely resulting from anxiety and school-based trauma: “I have left one job because of this and can’t take work that isn’t flexible as every day is not guaranteed she’ll attend school” (P284). For some, this was a “massive financial burden” (P69): “I lost my job and I am struggling financially. I am a single parent so the weight of this was all on my shoulders 24/7” (P140).

1. **“The devastating impact on us as a family”: The knock-on effects on the whole family.**
***“We’re always walking on eggshells”: Living in a ‘tense’ home***

Numerous parents used the phrase “walking on eggshells” to describe their situation at home. Parents often felt that their autistic child’s distress manifested as anger, aggression, or ‘meltdowns’, which eventually impacted the whole family: “walking on eggshells all of the time in case myself or family say or do the wrong thing and trigger his anger, flight mode, anxiety etc... it impacts everyone” (P211). Parents described feeling “tense” (P220), engaging in “constant battles” (P38), and noted how the “stress [was] leading to arguments” (P211). This was particularly difficult for other autistic family members: “there is a knock-on effect on myself and his sister as our ‘environment’ becomes more unpredictable too” (P263). Ultimately, they noted how their child’s difficulties had become difficulties that the whole family experienced: “we have all struggled alongside him” (P144).

In particular, the heightened levels of distress had an effect on the other siblings in the home, with respondents describing how siblings “had to hide when child was distressed/having meltdowns” (P14), found the “noise level difficult to deal with and struggles to understand how a situation can go from calm to almost an eruption over seemingly nothing” (P239), and found the “heightened emotions in the house very difficult to cope with” (259). Some had also experienced violence or aggression directed at them: “our youngest son suffered as a result of our eldest sometimes taking his anger out on him (P43); for some, this had resulted in their other children moving out or wanting to leave the family home: “our daughter throws heavy objects at us and hits us all or constantly screams. Our 17-year-old son no longer wants to live in his home that has become a war zone” (P273).

Furthermore, some reported how it had “affected whole family’s mental health” (P187), with one participant commenting “I can’t begin to tell you the devastating impact on us as a family” (P139). They explained how siblings were experiencing “low mood” (P143) and how it had “made younger brother anxious” (P187), often due to worry for their siblings, not understanding what is happening, and witnessing ‘meltdowns’. One parent explained the “huge impact on my other son who found suicide notes” (P228).

2.2. ***“We are as a couple struggling”: Strained relationships***

Parents noted that their relationships with others had become strained in numerous ways. One of the primary difficulties parents faced was the “massive toll” (P214) on their relationship with their spouse/partner: “we are as a couple struggling to stay together” (P165). Difficulties were often attributed to the loss of time to spend together when caring for their child without formal support: “me and my husband have not had an evening together in 10 years because bedtime routine is so time consuming and stressful” (P16) Parents also reported experiencing heightened emotional states and managing an overwhelming number of concerns, which resulted in intense discussions and conflicts: “arguments between parents due to worry” (P290). Several commented that they had since split up or divorced, with one parent noting: “[it was] devastating. My partner of 7yrs had to move out and I had to give up everything that mattered in my life” (P262).

Another significant area of concern for parents was their relationship with their other children. Many commented how their other children were receiving less attention or were “sidelined” (P73), due to the need to care for their autistic child’s mental health difficulties: “his sister was completely neglected while we ploughed everything in to keeping him safe” (P160). Parents felt that this lack of attention was negatively impacting their other children, including losing out on attention and activities: “siblings are effected in multiple ways by having to wait for my attention or they are unable to go places” (P84), and having to take care of themselves: “my daughter’s siblings have become carers and have to help each other get dressed and fed before school as all adult attention is on my daughter” (P83). Some also felt that this lack of attention was noticed, with siblings “often comment[ing] that my other daughter gets much more attention” (P110) and expressing feelings of resentment: “younger children notice and try to understand but sometimes the accommodations made to our child are resented” (P226). There were particular concerns for some parents that their other children might also be autistic but, because they were “functioning enough”, they were not “getting the attention they deserve” (P161):

“We have to spend a great deal of time with the 5-year-old to help regulate. We are unsure yet if the younger child will be ASC but we suspect, but he isn’t so aggressive so less time is spent with him which feels terrible.” (P165)

Parents also commented how caring for their child’s mental health needs while waiting for support had impacted their relationship with other members of extended family. They described how they were “currently seeing less of extended family” (P194) and their relationships with family members had been negatively impacted as they were “trying to help but not understanding” (P167). Parents felt that they were “isolated… from wider family” (P40) as they “cannot have family gatherings with child” (P93) and that caring for their child “prevents [them] participating in family events (P33)”. Some also commented how “family don’t like to visit” anymore (P192).

3. **“Isolated, alone, exhausted, and unsupported”: A lack of support**
  3.1. ***“Like a prisoner at home”: Loneliness, isolation, and stigma***

Many parents reported “feeling lonely” (P103) and experiencing “social isolation” (P297), with strained familial relationships (outlined above) and the loss of friendships a frequent concern. A common reason given for these feelings was that their autistic child would not leave the house during periods of mental ill health, largely due to anxiety/depression, which prevented the parents from socialising: “if your child won’t go out and is self-harming, you become separated and like a prisoner at home, as you have to look out for them, so it impacts on social life” (P75). Many parents also described how this had impacted the extent to which they could engage in activities as a family: “my partner and I have to take it in turns to leave the house… We can’t do things or go to places other families can, as a family. Extremely rare that we can go for a family day out together.” (p95). Other siblings were particularly affected and had to “cope with the changes” (P244), with them “miss[ing] out on lots of things as we can’t go out very often” (P95).

Parents explained that they had to reduce the number of activities they could engage in or places they could visit, “we cut back on a lot of activities” (P43), and they avoided new activities, “it makes everything more complicated as I try not to add anything new into our lives” (P282), or holidays “unable to go out anywhere in public or go on short breaks or holidays” (P255). Some parents commented that they “can’t plan to go anywhere until the day” (P102), while for others it meant that even if they did intend to go out, they often had to adjust their plans: “days out are restricted or cut short due to her emotional state and anxiety” (P120). As a result, parents described spending “a lot of extra time” planning (P270): “our world is much smaller as a result of our son’s anxiety. We don’t go out without careful planning” (P259). Parents who often had to cancel plans at short notice, described how this led to them losing friends:

“We have lost friends as we can’t stick to plans and are often late. We sometimes have to let people down at the last minute if she gets overwhelmed and can’t cope. We feel isolated and it also impacts on my mental health.” (P284)

Parents generally felt that others “don’t understand” what they were experiencing (P26), which led to them feeling “isolated, alone, exhausted and unsupported” (P273), and that they themselves no longer wanted to socialise due to their own deteriorating mental health: “I am now a pretty anxious and depressed person and so is my husband. Have withdrawn from most of my friends” (P95).

Another key perceived cause of isolation for several parents was stigma within the local community. This was typically described as implicit, often due to other people not understanding their child’s needs: “it makes us incredibly isolated and puts us on the periphery of local community due to the severe lack of understanding of her needs and how she presents behaviourally” (P245). One parent noted how this implicit stigma caused them to feel lonely:

“This [impact on the parent] is never talked about enough. It has turned my expectations for my life as a mother and my elder son’s life 180 degrees… The worst thing is how we are treated by other parents and the sympathy glances/horrified looks or mutters under their breath. It’s a lonely life as a parent of a special needs child.” (P70)

However, a minority did also describe explicit stigma: “the family have been subject to a great deal of violence and verbal cruelty” (P194), with one parent noting how their child’s experiences of bullying at school had led to the family becoming isolated in their village:

“I have become more insular and socially isolated as my social circle was centred around parents and children who attended the village school. As bullying in school was the beginning of our child’s anxiety issues, other parents have disassociated themselves from us as they don’t want to recognise that their children have been part of the problem.” (P150)

3.2. ***“Constantly overwhelmed and desperate for help”: The battle for more effective support from services***

Ultimately, all of the difficulties and challenges cited by the parents were felt to be a result of their child having mental health needs that were not being adequately addressed, with “no services at all, health or education, offering meaningful support” (P139). School was also commonly cited as a traumatic rather than supportive environment for many CYP, contributing to the onset of their mental health difficulties: “they had a traumatic event linked to school and their autistic needs not being met. This led to an acute episode of anxiety, this became chronic anxiety. That anxiety now makes every day life incredibly stressful for him” (P259). One parent described the lack of support from school and other services:

“I have been fighting for support at school and still haven’t got it... I have been trying to get CAMHS help but still haven’t got it. I’ve tried private psychiatric help. I have tried mind and parenting courses. I can’t go out. I can’t have friends visit. Family don’t like to visit. There is no respite. This is not what raising a child should be like and having a child try suicide at 12 is unbelievable and should not have got that far.” (P192)

Indeed, many parents described having to “fight” and “battle” for their child to get suitable support, explaining that trying to navigate the systems was “worse than you could possibly imagine” (P139). One parent described the negative impact the lack of support had on their own wellbeing:

“I’ve had to push for everything and deal with so many different services that I don’t know where to turn and feel hopeless much of the time. I’m a resilient and confident person, however going through this with no guidance has been hell.” (P284)

Feelings of hopelessness or powerlessness were common among parents, as they had to fight for help while also taking care of their unwell child: “there is the emotional stress of having a child that is unwell, trying to fight to get support for her from an overwhelmed and broken system and not knowing what the future holds for her” (P127). Many struggled to watch their unwell child not get the help they needed: “knowing that she is struggling and that I can’t get her the help she needs and deserves is heartbreaking” (P103). Some also felt blamed by professionals, which impacted their own mental health:

“School and professionals seem to only blame me and a lack of ‘boundaries’ for attendance issues. Struggle to find support for child and myself as a parent. My mental health has taken a battering as I am made to feel at fault, even though I am fighting to get support for my child. No information or support is freely given, everything is a fight. It is incredibly tiring and draining, as I just want what is best for my child but barriers are everywhere.” (P142)

A common issue was that parents felt the difficulties they faced were a result of professionals not understanding autism, meaning there were delays in accessing support:

“Substantial engagement with GP before a referral to CAMHS was made. CAMHS triage was awful. Little to no understanding of what the child was going through and therefore needed - particularly any knowledgeable responses provided given his likely autism… more waiting, more delay, more paperwork - a distressed child and very little if any real support at all. Exhausted.” (P168)

Some parents felt they themselves had to educate professionals by redressing gaps in their knowledge of autism and co-occurring conditions: “fighting for my daughter to get the support and understanding she needs in school for years. Trying to educate the schools, teachers, doctors, her mother, about autism and ADHD and getting absolutely nowhere!” (P264).

In particular, a lack of support from the child’s school was a source of stress and distress for parents. As above, parents felt some of the barriers were a result of schools not “having any understanding of the issues” (P39), meaning that parents were encountering avoidable difficulties including: “meeting the school several times a week about avoidable problems, things like our son being unlawfully excluded because the school did not meet his needs” (P30). One parent described how they felt their child’s school added to their worries:

“Fighting the school who are threatening me with fines [for absences]. CAMHS are supporting the school, not my daughter. It’s exhausting. There is no support from any of the people who purport to help or support us, there is just pressure to do what they want, not what my daughter needs.” (P178)

Another noted the impact this had on them on a daily basis: “it makes every school morning a point of worry. I feel constantly overwhelmed and desperate for help. I feel under huge pressure and this impacts us every day” (P250).

In some cases, the issues went beyond the school to the Local Authorities, with parents describing their children not being able to attend school at all: “had to spend a year battling the council for education” (P232). Several commented that they were going through, or had previously been to, a tribunal to get their child a place in school: “two years later our child is still on roll but almost entirely out of school, despite a diagnosis, medication, and EHCP [Education, Health and Care Plan] (no needs being met, tribunal pending)” (P241). One described the lack of support that was available for this process:

“I have fought to have his EHCP changed numerous times to try get the support he needs and I’m now fighting the local authority to amend it… This has also taken a lot of research and time on my part as there is no support or direction in terms of what help is actually available to parents and children.” (P170)

## Discussion

This is one of the first studies in the UK aiming to explicitly focus on understanding the experiences of parents supporting their autistic child with mental health difficulties. Findings illustrated the negative effects on parents’ mental health, with high levels of stress, worry, and exhaustion, and a poorer quality of life. Akin to previous research internationally [12,15,22,23], parents also highlighted the loneliness and isolation they felt, often as a result of stigma, as well as the wider ramifications on their family and their relationships. Ultimately, findings reflect the high levels of unmet need for support for both autistic CYP and their parents, with long waiting times, high thresholds for assessment in CAMHS, and an absence of appropriate support services resulting in a lack of improvement, or deterioration, in CYP’s mental health difficulties. As a result, parents were left to carry the significant burden of trying to keep their child safe from harm and supporting them at home in the best way that they can. In other words, they were having to act “in loco therapist” alone, to the detriment of their own and their family’s wellbeing. Similarly, parents reported their child’s school as a key issue, with their child’s mental health difficulties often beginning or being exacerbated there, due to unmet needs and neurotypical models of provision, along with a lack of help and support from the school when difficulties arose. Indeed, neurodivergent students face twice the emotional burden of their peers when navigating mainstream secondary schools [24], with upsetting experiences in education accumulating over time, shaping autistic students’ sense of belonging, self-worth and mental health [25].

The concerns that parents highlighted align closely with findings from the only other UK-based study conducted on this topic [17], with the inaccessibility of services, long delays, and a lack of professional knowledge about autism all negatively influencing CYP’s and parents’ experiences. Concerningly, this work was conducted 15 years ago, suggesting that little may have changed regarding mental health support for autistic CYP. Indeed, many of the issues raised by parents in the current study could be reduced if their children had access to timely and effective mental health support, both through healthcare services and in school. Parents described having to ‘fight’ to get their child support, and that the support they received was not adequate. This mirrors a recent report from Smith [26] which suggested that autistic CYP felt the support they received from CAMHS was ineffective, as it often centred around identifying symptoms and deficits rather than addressing the root causes of problems, and anxiety and autism were considered by professionals to be synonymous. CYP felt that CAMHS needed to offer appropriate and effective therapies, and that there needs to be a greater understanding of autism within services. Findings also align with existing evidence which highlights how parents can feel blamed by professionals for their autistic child’s mental health difficulties [27]. There is a therefore a clear need for a family-centred, sensitive, and neurodiversity-informed approach in CAMHS, which could include recognising the importance of supporting all family members (including siblings), personalising therapy adaptations for autistic individuals, and implementing strategies for different sensory and communication profiles [28,29]. It is also important for practitioners to acknowledge the minority stress and adversity as well as strengths of autistic CYP and their family members [30,31]. However, there is currently little robust evidence regarding the utility of adapted/alternative therapies for autistic populations – further research is needed in this area.

Furthermore, there is a need to explore the potential for an experience-driven model of peer coaching and mentoring in family hubs and CAMHS, to strengthen epistemic trust, increase social connectedness, and provide accurate and timely information that could help prevent crises for parents and carers of autistic CYP experiencing mental health concerns. Equally, it is important for CAMHS to work collaboratively with parents and participation groups, learning directly from their experiences about how to make services more accessible, responsive, and accountable to parents’ needs. Professionals working with autistic CYP and their families may also benefit from further neuroaffirmative training to ensure all have a clear understanding of what autism is (and what it is not), how mental health difficulties might present in autistic CYP, and reasonable adjustments that could be implemented. This is particularly pertinent given the current NHS Long Term Plan. One relevant initiative in England is the National Autism Trainer Programme (NATP), developed and commissioned by NHS England to equip professionals with the knowledge and confidence to provide more accessible and autism-informed care [32], which includes improving understanding of the needs of autistic people. With appropriate training, healthcare and education professionals may be able to intervene earlier to prevent mental health difficulties from developing, and they could provide more appropriate support for autistic CYP. This would in turn reduce the need for parents to provide intensive and long-term mental health support for their child, and would help to prevent parents feeling blamed by professionals for their child’s mental ill health. An experience sensitive approach [33] to inclusion in school policies and whole-school approaches (e.g., proactively and flexibly supporting students’ individual strengths and needs, making adaptations, sensory adjustments, alternative schooling and attendance options) may also reduce the likelihood of autistic CYP experiencing trauma at school, thus decreasing school-based anxiety/avoidance and helping to prevent the onset of avoidable mental health difficulties. However, in order for professionals to be able to implement effective strategies, resources and funding are needed across services and systems.

Many parents described the complex and unclear systems they faced when trying to seek support for their child, with a great deal of time and energy being required to navigate them. This aligns with previous research [17,34] highlighting the inaccessibility of services and the lack of overall support available for autistic CYP. Clearer systems and more bespoke services are urgently needed for the CYP themselves, as well as for their parents and siblings. There was also an overall lack of support for autistic CYP and their families, leaving CYP’s mental health to deteriorate and parents left holding responsibility for their safety. While improving children’s mental health would also have a positive effect on their family, parents still need to feel equipped to support them in maintaining good mental health in the long-term. As many of the parents in the present study shared feelings of burnout and isolation, peer or social support services may also be beneficial, as well as access to respite. It may also be useful to offer therapeutic options to parents while their children are receiving support from CAMHS. Furthermore, our findings align with those of Pavlopoulou et al. [31,35], highlighting the whole family’s need for sleep and mental health support, as well as for educational professionals to also consider the experiences of siblings, particularly during times their autistic siblings are unwell.

Finally, findings here reflect a more general issue within CAMHS, as identified in other studies [36], with high thresholds for assessment and long waiting lists resulting in a reactive ‘wait-to-fail’ model, with CYP ultimately reaching crisis point. While this is not specific to autistic CYP, it can disproportionately negatively impact this group who are already vulnerable to health inequalities. Thus, as highlighted by Edbrooke-Childs and Deighton [37], there is a need for more funding and service improvements to enable earlier intervention, rather than waiting for difficulties to escalate. Not only would this benefit the CYP directly, but it would also reduce the pressure, stress, and distress experienced by parents who are trying to support their children and keep them safe as their mental health continues to worsen while waiting for appropriate help.

### Limitations

The current study has several limitations to be consider. Firstly, parents/carers in this study self-selected and so may not be representative of the population as a whole. Indeed, the parents who were more engaged with support services and online communities were more likely to have been targeted. It is also possible that those who had a more positive experience may not have felt the need to participate. Secondly, as the survey was online and only available in English, some parents/carers may not have had access to the necessary technology; thus, it is possible that the families who are most isolated were not reached. Thirdly, this study only captured the views of parents/carers, and not CYP themselves, meaning that some vital experiences may have been missed. Fourthly, limited demographic information was collected from parents, meaning that any disparities in factors such as income level, rurality, availability of healthcare services, and cultural backgrounds were not captured.

## Conclusion

This study underscores the significant challenges faced by parents supporting their autistic child when experiencing mental health difficulties in the UK. The findings highlight the profound emotional and physical toll on parents, including stress, exhaustion, and isolation, exacerbated by long waiting times, inadequate support services, school-induced emotional burden, and a lack of understanding of autism within mental health and educational systems. Parents were often left to manage their child’s mental health needs without sufficient professional support, leaving them with little quality of life while also holding the worry, risk, and responsibility of keeping their child safe. There is an urgent need for more neurodiversity informed, personalised approaches to supporting autistic CYP and their families, along with timely access to effective interventions. Greater awareness and training for healthcare and education professionals, alongside clearer support systems and improved service infrastructure, are critical in addressing these issues. By ensuring earlier intervention and reducing systemic barriers, both the mental health of CYP and the wellbeing of their families can be significantly improved, ultimately fostering better outcomes for all involved.
